# A Rare Case of Mycotic Aneurysm Due to Methicillin-Sensitive Staphylococcus aureus (MSSA) Bacteremia

**DOI:** 10.7759/cureus.40336

**Published:** 2023-06-12

**Authors:** Sriharsha Dadana, Sisham Ingnam, Anusha Kondapalli

**Affiliations:** 1 Internal Medicine, Cheyenne Regional Medical Center, Cheyenne, USA; 2 Internal Medicine and Infectious Disease, Cheyenne Regional Medical Center, Cheyenne, USA

**Keywords:** staph aureus bacteremia, infectious aortitis, atypical presentation, persistent mssa bacteremia, mycotic thoracic aortic aneurysm

## Abstract

Mycotic aneurysm is defined as an infection of the arterial wall either by fungi or bacteria. Although, a rare complication of infection, it is associated with high morbidity and mortality. We describe a 69-year-old female with a rare thoracic aortic mycotic aneurysm, with no clear source of infection and a predominantly atypical presentation, manifesting primarily as heart failure, at a rural community hospital. Our case also depicts the rapid development of aortitis and mycotic aneurysms. This case highlights the challenges in the diagnosis and management of this condition.

## Introduction

The term “mycotic” was coined by William Osler in his Gulstonian lectures [1]. Although the name indicates fungal etiology, the predominant infectious causes are bacterial organisms. The incidence of infected aortic aneurysms is about 1-2%, and the thoracic aorta is one of the least common sites [2]. *Staphylococcus *spp, *Streptococcus *spp, and *Salmonella *spp appear to be the predominant bacterial causes [3]. The incidence in Western countries is much lower than in Asian countries [2].

## Case presentation

The patient is a 69-year-old woman with a history of type 2 diabetes, hypertension, and hyperlipidemia, who presented to the outside critical access emergency room (ER) complaining of worsening nausea and vomiting, body aches, and bilateral hip pain. She had a syncopal episode a day prior. While in the ER, a trauma workup with computerized tomography (CT) imaging of the head, chest, and hip was negative. A CT angiogram chest was obtained given hypoxia and syncope, which was unremarkable and did not show any acute processes. She did have a low-grade fever (100° F) but otherwise was hemodynamically stable; therefore, blood cultures were drawn, and she was given doses of ceftriaxone and azithromycin. Her labs were significant for normal white blood cell (WBC) at 6.6 th/mm3 with neutrophils at 62%, elevated B-type natriuretic peptide (BNP) at 2750 pg/ml, and elevated troponin at 0.77 ng/ml (threshold < 0.04 ng/ml). The EKG did not show any acute ST or T wave changes. Due to the concern of NSTEMI and acute hypoxic respiratory failure, the patient was transferred to our hospital for further evaluation.

On arrival, her BP was 158/73 mmHg and HR was 76 beats per minute, and she was hypoxic needing 8L oxygen via a high-flow nasal cannula. Given acute hypoxic respiratory failure, elevated BNP and troponin, and a physical exam showing crackles on the lung exam, cardiology was consulted, and diuresis with furosemide was initiated, as acute heart failure was the likely cause of her symptoms. Antibiotics were not continued, given no source of infection. On hospital day 3, her oxygen requirement got progressively worse, and she had a possible syncope or seizure episode secondary to hypotension (BP 60/40 mmHg) and was transferred to the ICU. At the same time, we were notified that her blood cultures done at admission at the outside ER came back positive for methicillin-sensitive *Staphylococcus aureus *(MSSA). Infectious disease was consulted, and the patient was started on nafcillin while repeat blood cultures were ordered.

**Figure 1:**
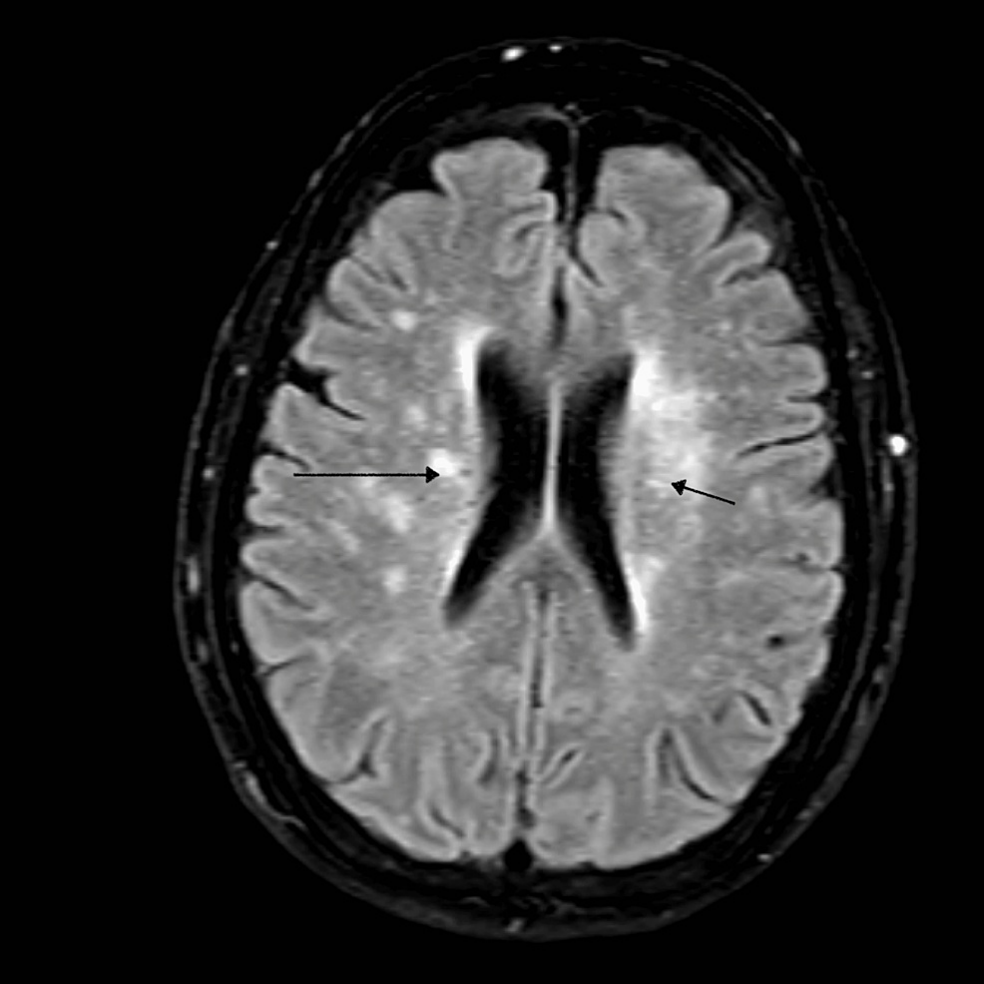
MRI Brain showing multiple embolic infarcts (arrows)

On hospital day 4, the repeat blood cultures came positive for MSSA with neg mecA/B and WBC uptrend to 19,000 /mm3. Magnetic resonance imaging (MRI) of the hip and spine was done for a source workup and was negative. Given the patient presented with syncope, an MRI of the brain was done, which showed the presence of multiple embolic strokes (Figure 1).

**Figure 2:**
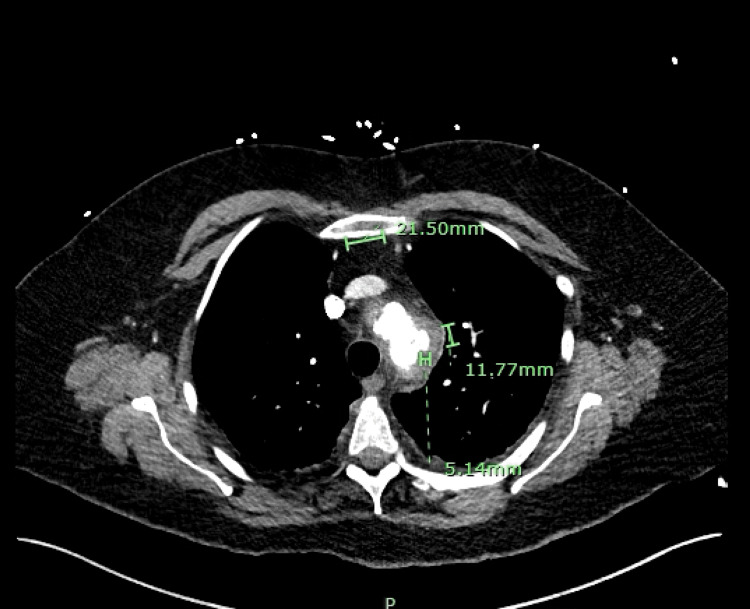
CT Angiogram Chest showing 0.5 x 1.2 cm eccentric hyperdense collection outside the boundary of the aorta

Trans-thoracic echo (TTE) and trans-esophageal echo (TEE) were done which did not show evidence of Infective endocarditis but did show evidence of diastolic dysfunction. Blood cultures repeated on hospital day 5 also came back positive for MSSA despite being on nafcillin for 48 hrs. Given persistent bacteremia despite being on nafcillin, repeat imaging of the chest was done and ertapenem was added for dual coverage. Repeat CT Angiogram Chest (Figure 2) now showed developing or increasing inflammatory stranding of the peri-aortic fat lateral to the top of the aortic arch. A 0.5 x 1.2 cm eccentric hyperdense collection was noted outside of the boundary of the calcified plaque of the aorta, findings consistent with extra aortic contrast of the top of the aortic arch with a differential diagnosis being mycotic aneurysm, ulcerative plaque, focal aortic rupture. She did not have any of these imaging findings on prior CT Angiogram Chest done on hospital day 1 in ER.

Given the rapid development of aortic aneurysm with aortic inflammation, and persistent positive blood culture with MSSA despite being on antibiotics, it was determined to be likely infective aortitis with mycotic aneurysm from MSSA bacteremia for which the patient needed urgent intervention. She was transferred to a tertiary center for cardiothoracic surgery intervention.

At the tertiary center, PET/CT was done which showed increased fluoro-deoxy-glucose (FDG) uptake localizing to a periaortic collection extending from the distal aortic arch into the proximal left subclavian artery compatible with suspected mycotic infection. Additional areas of intense curvilinear FDG activity localizing to the anterior wall of the descending thoracic aorta just above the celiac artery origin suspicious for additional sites of aortic infection were also seen. Also, uptake was noted in the thoracic vertebra and brain indicating likely septic emboli. Given the patient's clinical condition and inability to tolerate open repair, endovascular repair was done. She underwent Trans-catheter Endo-Vascular Aortic Repair (TEVAR) with a plan for indefinite antibiotic suppression with nafcillin intravenously initially for 6 weeks followed by lifelong suppression with oral dicloxacillin.

The patient was discharged from the hospital to rehab but later had recurrent admissions due to heart failure, pneumonia, and renal failure and deceased 3 weeks later. Follow-up repeat CT on readmissions showed a smaller area of opacification and a decrease in the size of the aneurysm.

## Discussion

Thoracic aortitis and mycotic aneurysms are rare in this antibiotic era [1,3]. An infected aneurysm is a localized dilation of an artery due to the destruction of the vessel wall by infection. The organisms most likely responsible are *Staphylococcus *spp and *Salmonella *spp. Other organisms include *Streptococcus pneumoniae*, *Treponema pallidum*, and *Mycobacterium tuberculosis*, as well as other bacterial, fungal, or anaerobic pathogens [3,4]. Infected aneurysms can occur in any artery, but the femoral artery has recently become the most common location of all infected aneurysms, followed in frequency by the aorta, intra or extra-cranial, innominate, iliac, and splanchnic arteries [5]. In non-endocarditis bacteremia, the abdominal aorta has been the most common site due to the predominant atherosclerotic plaque in this location [6,7].

Aortitis leading to an infected thoracic aortic aneurysm is exceedingly rare. The pathogenesis leading to an aneurysm is most commonly due to a septic embolism, but direct bacterial inoculation, bacteremic seeding, and contiguous infection can also occur [7-9]. The infection weakens the aortic wall’s constituents, leading to rapid aneurysm formation. Risk factors for infected aneurysms include trauma, endocarditis, impaired immunity with malignancy, HIV, diabetes, and advanced age group patients with atherosclerotic disease [9,10]. Healthy individuals are usually resistant to aortic infection. Like in our patient, diabetes and atherosclerosis disease pose a risk factor for bacterial inoculation.

One of the reasons for the delay in diagnosis is the paucity of symptoms. Most of the symptoms are nonspecific. Symptoms also depend on the site of infection and aneurysm formation. Fever, thoracic and dorsal pain, abdominal pain, and chills are the most common symptoms. Fever of unknown cause is also one manifestation. A high index of suspicion is necessary, as the most common course of the disease is fatality due to fulminant sepsis or massive hemorrhage if left untreated [11].

The diagnosis of an infected aneurysm is based on imaging the aneurysm, and infection is confirmed by culturing an organism. Leukocytosis and neutrophilia are present in 65-83% of cases. Most patients’ erythrocyte sedimentation rate and C-reactive protein level are elevated [2].

Blood cultures are positive in 50-75% of patients, with reduced rates in those already on antibiotics. Cultures taken from surgically resected infected tissue also aid in microbiological identification if peripheral cultures are negative [12-14]. A CT scan with contrast is the modality of choice to diagnose an infected aneurysm. Findings on CT angiography suggestive of an infected aneurysm include inflammation around the vessel, perivascular fluid, and aneurysm with intramural air [2,15-17]. PET-CT also has high sensitivity and is an important aid in diagnosing the extent of inflammation [17].

There are no randomized trials to guide the management of infected aneurysms. The standard treatment for most infected aneurysms is antibiotic therapy combined with surgical debridement. Antibiotic therapy is usually tailored to the organism identified and its sensitivities. The optimal duration of antibiotics is not well defined and should be tailored to individual cases and the choice of intervention. Surgical options include open resection and endovascular repair; however, the optimal surgical option remains controversial. Given our patient’s comorbid conditions and poor clinical status, endovascular intervention with TEVAR was pursued with a plan for indefinite antibiotic suppression [18]. For patients who have significant comorbidities and poor clinical status, thoracic endovascular repair may be an appropriate palliative option, given the even higher mortality associated with open surgery in this subset of patients.

## Conclusions

We describe a case of mycotic aneurysm in MSSA bacteremia. Our case elucidates the need for physicians to remain vigilant and consider this differential in the case of persistent bacteremia and a negative workup for endocarditis. Treatment options should be tailored to individual patient conditions. Early diagnosis and treatment are crucial, given the high morbidity and mortality with delayed diagnosis.
